# Gut microbiota components are associated with fixed airway obstruction in asthmatic patients living in the tropics

**DOI:** 10.1038/s41598-018-27964-3

**Published:** 2018-06-25

**Authors:** Emiro Buendía, Josefina Zakzuk, Homero San-Juan-Vergara, Eduardo Zurek, Nadim J. Ajami, Luis Caraballo

**Affiliations:** 1 Institute for Immunological Research, University of Cartagena, Cartagena, Colombia; 2 Department of Medicine, Universidad del Norte, Barranquilla, Colombia; 3 Department of System Engineering, Universidad del Norte, Barranquilla, Colombia; 4 Alkek Center for Metagenomics and Microbiome Research, Baylor College, Houston, USA

## Abstract

Microbiome composition has been associated to several inflammatory diseases, including asthma. There are few studies exploring the relationships of gut microbiota with airway obstruction pheonotypes in adult asthma, especially those living in the tropics. We sought to evaluate the relationships of gut microbiota with the airway obstruction and other variables of interest in asthmatic patients living in the tropics according to three phenotypes: No Airway Obstruction (NAO), Reversible Airway Obstruction (RAO) or Fixed Airway Obstruction (FAO). We found that *Streptococcaceae:Streptococcus* and *Enterobacteriaceae:Escherichia-Shigella* consistently discriminated asthmatic individuals suffering FAO from NAO or RAO, plus *Veillonellaceae:Megasphaera* when comparing FAO and RAO (p < 0.05; FDR < 0.05). In the FAO, the network showing the genus relations was less complex and interconnected. Several *Rumminococcaceae*, *Lachnospiraceae* and *Clostridiales* were enriched in patients with low specific IgE levels to mites and Ascaris. All patients shared a common exposure framework; control medication usage and smoking habit were uncommon and equally distributed between them. In conclusion, in this tropical asthmatic population, components of human gut microbiota are associated with the presence of a FAO phenotype and lower specific IgE response to mites and Ascaris.

## Introduction

Asthma is a chronic inflammatory respiratory disease affecting about 10% of humans. Although genetic predisposition is determinant for disease development, environmental factors also have a role. Microbial colonization of body sites may be shaped by both, host genetics and environment (i.e. diet and geography). Microbiome composition has been associated to several inflammatory diseases, including asthma^1–3^. There are an increasing number of publications that shows a link between intestinal or airway microbial composition and the incidence or severity of asthma in animal models and humans^4–9^. Different mechanisms derived from microbiome-host interactions and metabolites could explain how a microorganism could impact disease presentation^1,2,4,5^. Moreover, restoration of gut microbiota, through the use of prebiotics or probiotics, has shown positive results in asthma-related clinical outcomes^10^. Heterogeneity at different levels, personal and geographical, makes it difficult to identify microorganisms or microbial networks associated with diseases^11^. In the case of asthma, it is also recognized that it has different disease phenotypes^12^. Further profiling and characterization of the microbiome associated with different asthma phenotypes is necessary for identifying novel microbiota-related mechanisms of disease.

Other authors have found association of airway obstruction phenotypes with different immune traits and disease severity^13^. Bronchodilator response (BDR) is a classical asthma phenotype that means reversibility of airway obstruction after the administration of a short-acting Beta 2 agonist, a common relief medication for asthma symptoms. Although its patophysiology and relationships with clinical outcomes are partially understood, it is recognized as complex trait involving interactions of different cell types and genetical determinants^14^. Reversible airway obstruction is considered as a criterium for asthma diagnosis and it has been associated with biomarkers of eosinophilic inflammation, atopy and bronchial hyperreactivity^15–17^. Cluster analysis has suggested that the BDR is an important component in defining asthma phenotypes^18^. On the other hand, lack of airway obstruction reversibility (also known as fixed airway obstruction) is another phenotype of asthma found in some asthmatic individuals^19,20^.

In general, there are few studies about microbiome differences in regard to asthma phenotypes, but less information exist about microbiome composition of tropical populations, especially from Latin America^21,22^. The aim of this study was to evaluate the relationships of gut microbiota with the airway obstruction and other variables of interest in adult asthmatics living in the tropics.

## Methods

### Design, location and study population

This study analyzes the gut microbiome of asthmatic subjects included in the Ascaris and Asthma Severity program; which assesses the relationship between asthma severity and *Ascaris lumbricoides* sensitization^23^. Patients live in Cartagena de Indias in the North Caribbean coast of Colombia, a tropical city (10° 23′ 59″ North, 75° 30′ 52″ West) with around 1 million inhabitants, most of them poor according to the govermental indexes^24^. Socioeconomic stratification in Colombia ranges from 1 to 6, and the majority of the city population, as well as the study participants, belonged to the lowest strata 1–3 and shared environmental conditions. The genetic background resulted from racial admixture between Native Americans, Spaniards, and an important proportion (37.9%) of African ancestry^25,26^. The study was approved by the Ethics Commitee of the University of Cartagena (Cartagena, Colombia), all experiments were performed in accordance with and following the Declaration of Helsinki Principles. Signed informed consent was obtained from patients or their parents. Stool sample for microbiota analysis was collected from 202 subjects of the program.

### Eligibility criteria and enrollment procedures

Subjects attending five public primary health care centers and the University Hospital were screened for eligibility by physicians of the research staff between June 2010 and March 2011. These centers serve the lowest social strata in the city. Eligibility criteria were: subjects in the age range of 8 to 70 years who answered affirmatively to the question: *Have you ever been diagnosed with asthma?* Inclusion in the study depended on the confirmation of asthma diagnosis made by the physician. Control medication usage was defined as the regular use of oral (OCS) or inhaled corticosteroids (ICS) and evaluated by questionnaires. Patients with chronic obstructive pulmonary disease (COPD) or another chronic respiratory co-morbidity were excluded as well as those belonging to the highest socio-economical strata of the city (4 to 6).

### Asthma diagnosis

Eligible subjects were further interviewed and asthma diagnosis was confirmed in those with at least two respiratory symptoms (cough, wheezing, dyspnea, and nocturnal cough/wheezing/dyspnea) or a history of recurrent asthma attacks. These questions were done by staff physicians following a validated questionnaire^27,28^.

### Skin prick test

Skin prick test (SPT) was done in the forearm with a battery of allergen extracts (kindly supplied by Leti; Madrid, Spain) as previously described^23^. Atopy was defined as at least one positive SPT to any of the tested allergens.

### Assessment of lung function and the airway obstruction phenotypes

Spirometry was performed with a Microlab spirometer (Carefusion Corporation, San Diego, USA) following the American Thoracic Society recommendations^29^. Height and weight were measured; patients were instructed to avoid use of short-acting bronchodilators for at least 12 hours before testing. The best forced expiratory volume in one second (FEV1) was selected and was taken as an objective indicator of basal airway obstruction; bronchodilator response was defined as >12% improvement in the predicted basal FEV1 levels after 200 µg of salbutamol inhalation. According to spirometry results, subjects were classified into 3 different phenotypes: No Airway Obstruction (NAO: Symptoms + FEV1 ≥ 80% predicted; n = 66), Reversible Airway Obstruction (RAO: Symptoms + FEV1 < 80% + Bronchodilator Response; n = 74), Fixed Airway Obstruction (FAO: Symptoms + FEV1 < 80% predicted + Lack of Bronchodilator Response; n = 42).

### Quantification of total and specific IgE

In the tropics, helminthiases are common and house dust mites (HDM) exposure is perennial. We previously showed that Ascaris and HDM sensitization is associated with indicators of asthma severity^23^; therefore, we evaluated the relationship of specific IgE levels with microbiota composition. Briefly, blood samples were taken by venipuncture using anticoagulant-free tubes to obtain serum for antibody determinations. Serum total IgE and specific IgE levels against *B. tropicalis*, *D. pteronyssinus* and *A. lumbricoides* were determined by ImmunoCap system (Phadia100, Thermo, Sweden).

### Stool DNA extraction

Participants were asked to bring a recently collected stool sample in a hermetic recipient provided by the research team. Samples kept refrigerated less than 12 hours between collection and frozing at −20 °C until processing. Samples were thawed for the first time and DNA extraction was performed using Qiamp DNA stool minikit (Qiagen, Hilden, Germany) according to the manufacturer’s instructions. DNA concentration was quantified using a Nanodrop spectrophotometer (Thermo Scientific, Waltham, USA). Eluted DNA was stored at −80 °C until amplification reaction and V4 region sequencing.

### Amplification and sequencing of variable 4 (V4) region of 16S rRNA gene

Genomic bacterial DNA extraction methods were optimized to maximize the yield of bacterial DNA while keeping background amplification to a minimum. 16S rRNA gene sequencing methods were adapted from the methods developed for the NIH-Human Microbiome^30,31^. Briefly, the 16S rDNA V4 region was amplified by PCR and sequenced in the MiSeq platform (Illumina, San Diego, USA) using the 2 × 250 bp paired-end protocol yielding pair-end reads that overlap almost completely. The primers used for amplification contain adapters for MiSeq sequencing and dual-index barcodes so that the PCR products may be pooled and sequenced directly^32^, targeting at least 10,000 reads per sample.

Our standard pipeline for processing and analyzing the 16S rRNA gene data incorporates phylogenetic and alignment-based approaches to maximize data resolution. The read pairs were demultiplexed based on the unique molecular barcodes, and reads were merged using USEARCH v7.0.1001^33^, allowing zero mismatches and a minimum overlap of 50 bases. Merged reads were trimmed at first base with Q5. In addition, a quality filter as applied to the resulting merged reads and reads containing above 0.05 expected errors were discarded.

16S rRNA gene sequences were assigned into OTUs or phylotypes at a similarity cutoff value of 97% using the UPARSE algorithm. OTUs were then mapped to an optimized version of the SILVA Database^34,35^ containing only the 16S v4 region to determine taxonomies. Abundances were recovered by mapping the demultiplexed reads to the UPARSE OTUs. A custom script constructed an OTU table from the output files generated in the previous two steps, which was then used to calculate alpha-diversity, beta-diversity^36^, and provide taxonomic summaries that were leveraged for all subsequent analyses discussed below.

### Parasitological examination

Parasitological analyses were done using 0.85% saline solution and lugol staining; counting helminth eggs were done by the Kato Katz method using a commercial kit (Copro Kit, C&M Medical, Campinas, Brazil). The presence of eggs from geohelminths or parasite visualization was considered diagnostics of active infection.

### Statistical and network analysis

In socio-demographic analyses, differences between proportions were analyzed by Pearson chi-squared test (or Fisher exact test when appropriate). Total and specific IgE values were not normally distributed and they were therefore reported as the median value and its interquartile range, except total IgE (geometric mean). Kruskal-Wallis (KW) test was used for comparison of continuous variables among three groups. *Post hoc* analyses were also performed.

Analysis of microbiota datasets presented several technical challenges. First, OTU abundances are compositional and not independent, thus conventional comparison or correlation between OTU abundances using traditional statistical methods can lead to spurius results. Secondly, variables in an OTU table outnumber the tested individual samples, thus inference of OTU-OTU association networks is underpowered^37^. Analyses were done using R scripts and to reduce false positive, only OTUs with at least 1 count in 50% of samples were included in the analysis. The compositional nature of the data, characterized by non-negative counts whose weight is relative to the other components of a given sample, restricts the analysis of the data to a simplex space. In order to carry the data to a normal space, a Centered Log Ratio Transformation (CLR) of Aitchinson was applied, this transformation was done using the function *logratio.transfo* of the mixOmics package^38^. Since this is a logarithmic transformation, it requires a pre-conditioning of the data to replace the zeros, this substitution was performed with a multiplicative Bayesian replacement strategy using the *cmultRepl* function of the zCompositions package^39^.

Alpha diversity was calculated using Chao 1 and Shannon indexes using the Phyloseq package^40^. sPLS-DA used an approach that asks and identifies which features (OTUs) separates patients according to airway obstruction phenotypes based on a discriminant analysis of the partial least square metric. To select the most predictive/discriminative OTUs classifying the samples according to the airway obstruction phenotypes or specific IgE levels, Sparce Partial Least Square Linear Dyscriminant Analysis (sPLS-DA) was done using the package Mixomics including 10 variables in the first component^38^. The Percentile 25^th^ and 75^th^ were calculated to select the extremes of the distribution of specific IgE levels to *Ascaris, B. tropicalis* and *D. pteronyssinus* in order to discriminate between high IgE responders (HR, levels ≥ percentile 75^th^ to the highest) and low IgE responders (LR, levels ≤ percentile 25^th^). Since percentile 25^th^ values were 0,2, 0,2 and 0,1 kUL for Ascaris, *B. tropicalis* and *D. pteronyssinus* – specific IgE, respectively, all patients included in LR were not sensitized to that specific source. Percentile 75^th^ values were 3,1, 23,7 and 13,03 for Ascaris, *B. tropicalis* and *D. pteronyssinus* – specific IgE, respectively. Thus, those in HR group were sensitized individuals with the the highest responses.

As individuals living in the tropics are exposed to Ascaris infection and this could alterate the gut microbiota, sPLS-DA was also done using Ascaris infection status as a binary category. Iterative cross validation method allowed us to select the most stable OTUs in the model of classification; wich means those OTUs always indicating differences in the individuals belonging to the category of interest after iterative comparison with the individuals of the other category. To avoid a type 1 error after the selection of the most consistent OTUs (Stability Index = 1), row counts were later compared between phenotypes of inteterest using the Metagenomeseq package normalizing the data with Cumulative Sum Scaling by applying the cumNorm function and performing the analysis with a zero-inflated log-normal model by applying the fitFeatureModel, this package employ the Wilcoxon signed-rank test and posterior correction for multiple comparisons with the Benjamini-Hochberg method^41^. Due to significant differences in age among phenotypes, the influence of age as a continuous variable on OTU counts was evaluated the MaAsLin package^42^.

To predict OTUs networks’ interactions within each asthma group, undirected conditional independence network graphs were constructed using Sparce and Compositionally Robust Inference of Microbial Ecological Networks (SPIEC-EASI) statistical method^37^, which used the Meinshausen-Buhlmann strategy as the graph estimator. Finally, differentially enriched OTUs, detected by sPLS-DA, were localized into the predicted interaction network to find nodes and clusters of interest differentiating the groups.

## Results

### Characteristics of the study population

Twenty out of 202 sequenced samples were excluded from the analysis because OTU counts were too low (n = 3) or due to lack of spirometry data^23^. The V4 region of the 16S rRNA was amplified and sequenced in all of them obtaining at least 10.000 sequencing reads per sample (see Supplementary Fig. S1,2). Sociodemographic and clinical features of the study population are shown in Table 1. Overall, we included asthmatic patients sharing an environmental exposure framework. NAO patients were younger than those with obstruction. Specific IgE levels to *D. pteronyssinus* were different among phenotypes, being higher in RAO patients than those with FAO.

**Table 1:** Socio-demographic characteristics of the study population.

Variables	All patients (n = 182)	FAO (n = 42)	RAO (n = 74)	NAO (n = 66)	p-value
Gender (male)	23.6	21.4	18.9	30.3	0.26
Age (mean ± SD)	33.7 ± 17.3	36.5 ± 17.5	39.4 ± 16.9	25.4 ± 14.3	<0.0001
Socio-economical strata					0.32
1	63.18	64.28	68.91	56.06	
2	29.12	28.57	21.62	37.87	
3	7.69	7.14	9.45	6.06	
Smoking habit	2.74	2.38	4.05	1.51	0.64
Co-habitation with a smoker	25.27	33.33	25.67	19.69	0.26
Predicted baseline FEV_1_ (%)	71.87 ± 21.47	66.03 ± 12.29	56.63 ± 18.79	92.50 ± 8.67	<0.0001
Predicted baseline FEV_1_/FVC (%)	86.57 ± 15.44	88.04 ± 14.56	77.14 ± 15.41	96.5 ± 7.65	<0.0001
Episodes of severe dyspnea	66.48	69.04	71.62	59.09	0.27
Nocturnal awakenings	87.36	79.57	93.24	86.36	0.06
>4 ER visits int the last year	26.37	23.80	35.13	18.18	0.07
Hospitalizations in the last year	13.73	23.80	13.51	7.57	0.06
Bronchodilator responsiveness	12.63 ± 19.40	2.45 ± 6.90	27.95 ± 21.92	1.93 ± 5.29	<0.0001
Use of oral corticosteroids	30.81	26.31	35.71	28.12	0.50
Use of inhaled corticosteroids	30.81	26.31	35.71	28.12	0.5
Current Ascaris infection	4.39	4.76	5.40	3.03	0.81
Antecedent of worm expulsion	60.43	66.66	62.16	54.54	0.43
Allergic rinitis	84.61	88.69	83.78	83.33	0.80
Atopy (>1 positive SPT)	83.51	78.5	86.4	83.3	0.54
Sensitization to *B. tropicalis*	64.20	59.52	69.53	61.53	0.46
Sensitization to D. Pteronyssinus	69.88	64.28	76.81	66.15	0.27
Total IgE^‡†^	654.6695 ± 602.8	525.70 ± 979.54	826.76 ± 1767.43	543.78 ± 637.61	0.09
Specific IgE to *B. Tropicalis*^†^	19.52 ± 33.54	20.31 ± 30.39	19.42 ± 33.60	19.12 ± 35.79	0.6
*Specific IgE to D. Pteronyssinus* ^†^	19.12 ± 36.61	11.02 ± 24.51	27.08 ± 44.70	15.36 ± 31.32	0.01
Specific IgE to *A. lumbricoides*^†^	3.82 ± 2.745	2.82 ± 6.51	4.29 ± 8.4	3.93 ± 9.41	0.07

^†^Comparison by Krukal-Wallis test.

Frequency rates (%) for categorical variables are shown.

^‡^Geometric mean (standard deviation of mean) are reported.

### Gut microbial richness and diversity was similar among airway obstruction phenotypes

**Figure 1:**
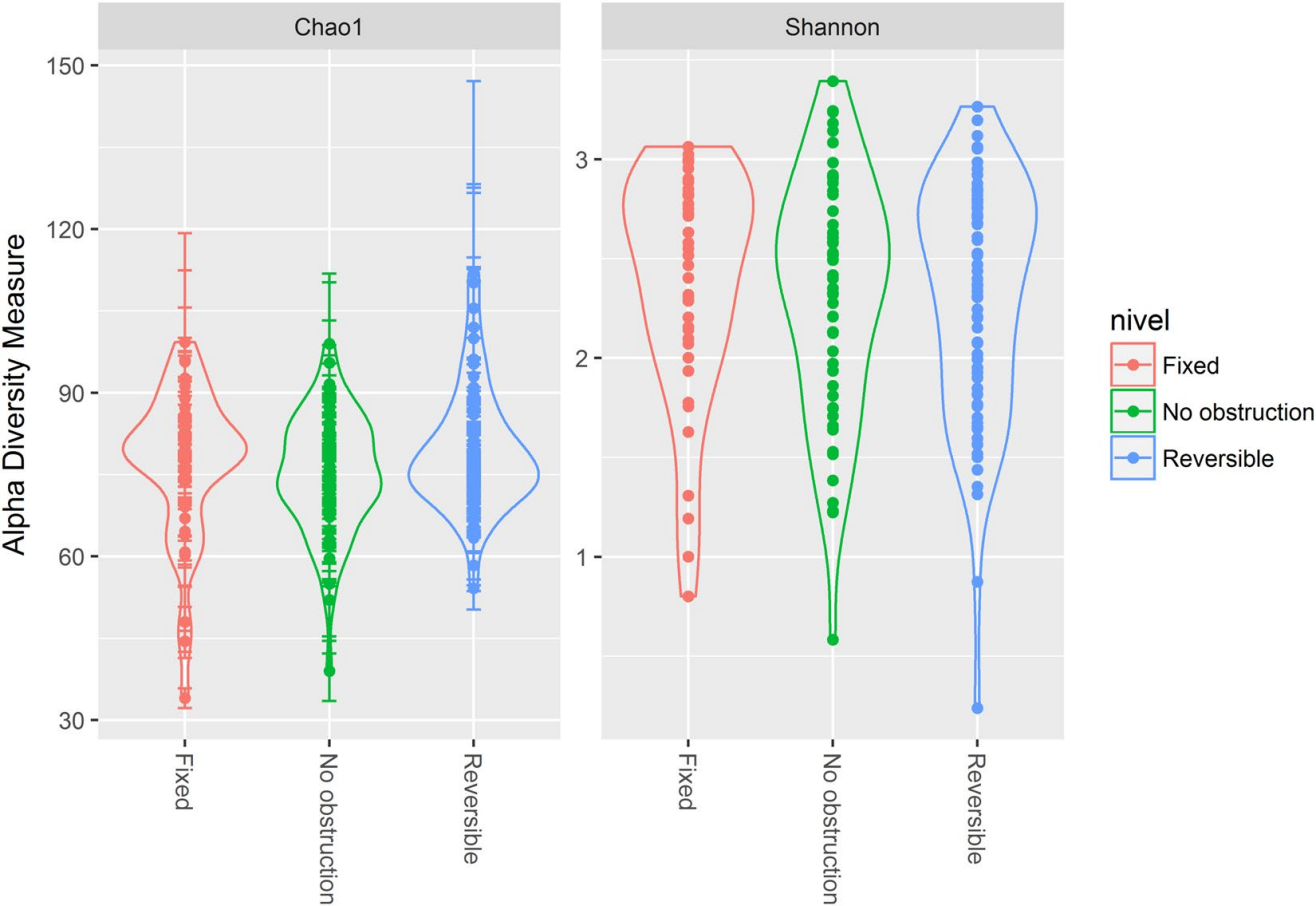
Comparison of alpha diversity among different airway obstruction phenotypes using Chao 1 (**A**) and Shannon diversity indexes (**B**). Violin plot includes the median, 95% CI, IQR, and density plot where the width of the differently colored lines indicate frequency.

Microbial richness and diversity were evaluated by means of the Chao 1 and Shannon indexes, respectively. Based on the OTUs distribution, the average value of Chao 1 was not different between phenotypes, showing the following mean values: 76.8 ± SD 13.91, 79.1 ± SD 11.25 and 75 ± SD 11.02 for FAO, RAO and NAO phenotype, respectively (KW Test; p = 0.25; Fig. 1A). Mean Shannon index values were neither different among groups: 2.37 ± SD 0.58, 2.31 ± SD 0.59 and 2.32 ± SD 0.59 in asthmatics with FAO, RAO and NAO, respectively (KW test; p = 0.79; Fig. 1B).

### *Streptococcaceae:Streptococcus and Enterobacteriaceae:Escherichia-Shigella* consistently discriminated and were enriched in patients with the FAO phenotype

**Figure 2:**
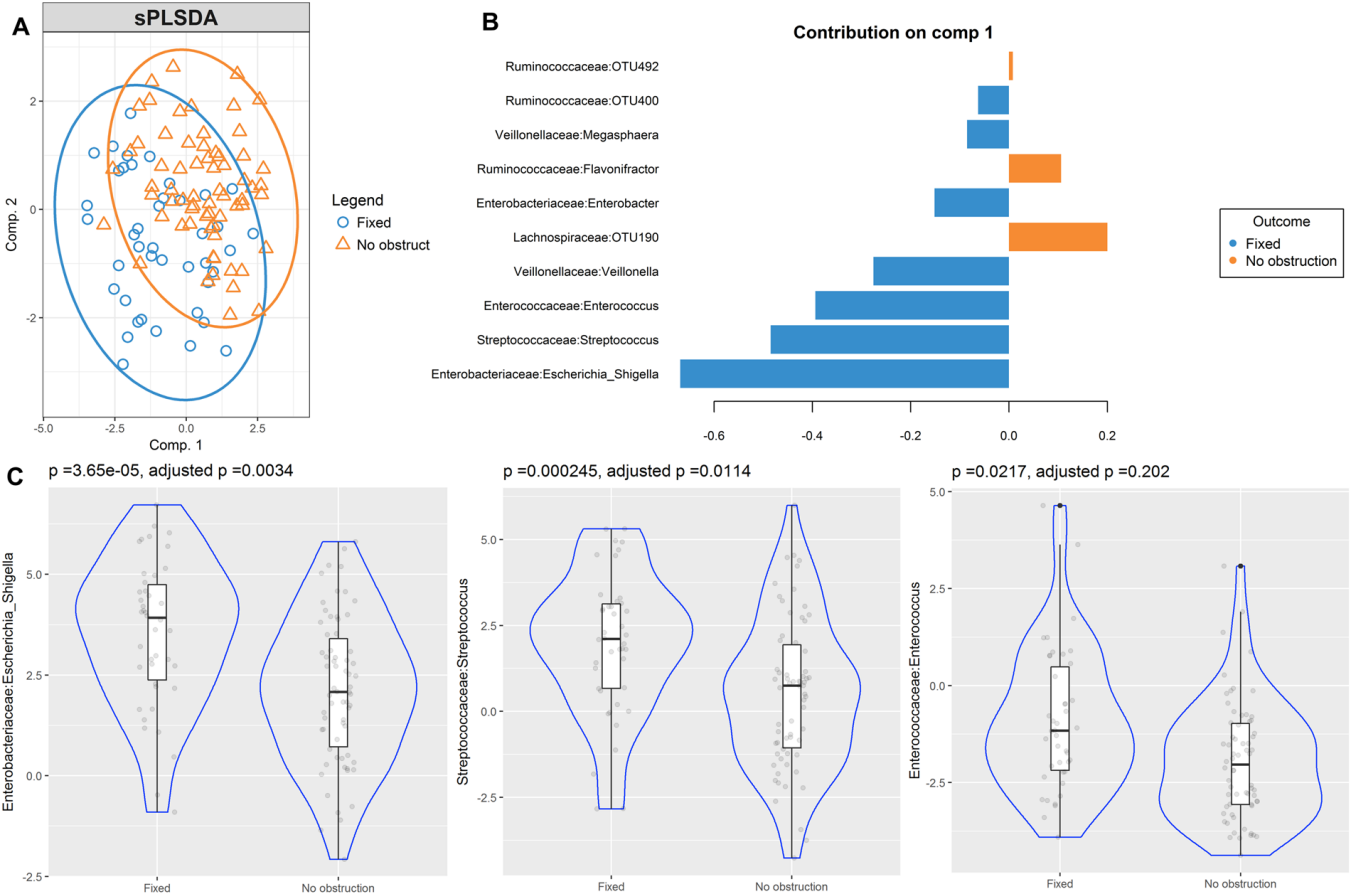
Partial least squares discriminant analysis of gut microbiome composition between patients with fixed airway obstruction versus no obstruction sPLS-DA plot based on the relative abundance of bacterial taxa of gut microbiota from patients with fixed airway obstruction (blue circle) or without obstruction (orange triangle) and their 95% confidence ellipses (**A**). Contribution plot indicating genera contributing to component 1 of the sPLS-DA plot that discriminate these phenotypes (**B**). The abundance of the most consistent OTUs was compared using Metagenomeseq and presented on a violin plot, which includes the median, 95% CI, IQR, and density plot where the width of the blue lines indicate frequency (**C**).

**Figure 3:**
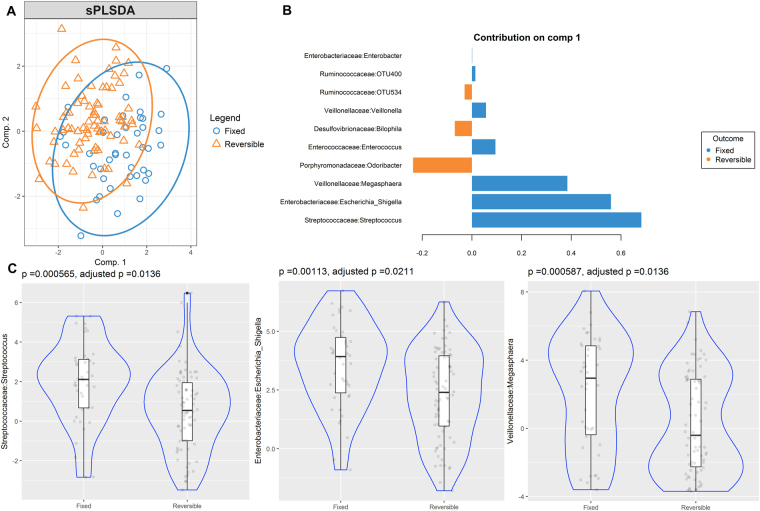
Partial least squares discriminant analysis of gut microbiome composition between patients with fixed versus reversible obstruction. sPLS-DA plot based on the relative abundance of bacterial taxa of gut microbiota from patients with fixed airway obstruction (blue circle) or reversible airway obstruction (orange triangle) and their 95% confidence ellipses (**A**). Contribution plot indicating genera contributing to component 1 of the sPLS-DA plot that discriminate these phenotypes (**B**). The abundance of the most consistent OTUs was compared using Metagenomeseq and presented on a violin plot, which includes the median, 95% CI, IQR, and density plot where the width of the blue lines indicate frequency (**C**).

sPLS-DA analyses were used to identify OTUs associated with airway obstruction phenotypes. As observed in the bi-plot, microbial composition showed a clear differentiation between patients with FAO and NAO (Fig. 2A). Several differentially abundant OTUs in FAO and NAO stool samples that contributed to their separation in component 1 were identified (Fig. 2B). *Enterobacteriaceae:Escherichia-Shigella, Streptococcaceae:Streptococcus* and *Enterococcacea:enterococcus* were enriched in FAO patients and accurately discriminated them from those with NAO. These bacteria were also the most consistent OTUs (Stability index = 1), being present in 90.9%, 98.8% and 56.7% of samples, respectively. After correction by multiple comparisons, only *Enterococcus:Escherichia-Shigella* and *Streptococcaceae:Streptococcus* showed significant enrichment (Fig. 3C and Supplementary Table S1). Microbial composition of patients with FAO was also clearly discriminated from those with RAO. *Streptococcaceae:Streptococcus* and *Enterococcus:Escherichia-Shigella* plus *Veillonellaceae:megasphaera* were significantly enriched in FAO and were the most consistent taxa (Stability index = 1) and (Fig. 3A,B). Furthermore, after correction by multiple comparisons, all of them kept significantly enriched (Fig. 3C and Supplementary Table S1). In addition, none of the above was significantly associated with age and remained discriminant after removing patients younger than 18 years in sensitivity analysis with sPLS-DA (data not shown). The sPLS-DA analysis showed that in females the same OTUs remained discriminant, but in males only *Enterobacteriaceae:Escherichia-Shigella* remained discriminant (see Supplementary Figs S3,4). However, as we found no differences on male proportion between phenotypes (Table 1), it seems that gender distribution is not a confounding variable. Comparison of the two other phenotypes (NAO vs RAO) did not let to significantly enriched taxa after adjustment (see Supplementary Fig. S5 and Table S1).

### FAO phenotype harbour a distinctive network of OTUs

**Figure 4:**
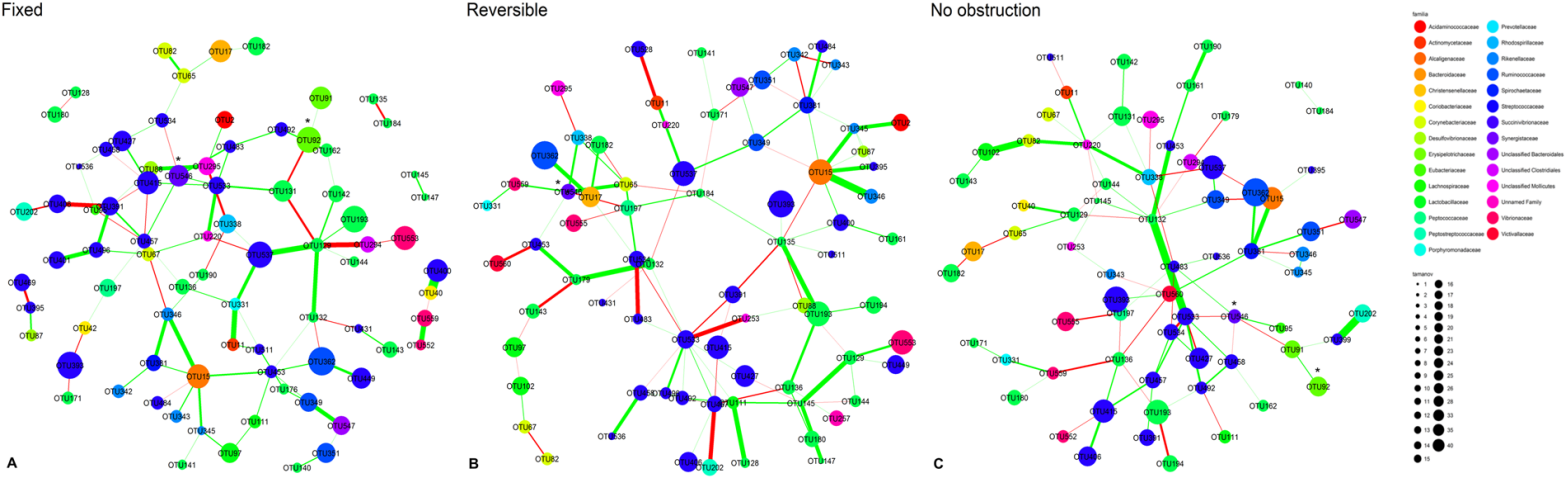
MB Networks from patients with fixed (**A**), reversible (**B**) and no airway obstruction (**C**) using SPIEC-EASI method. Overall, networks display a differential association pattern between OTUs according to airway obstruction phenotypes. Consistently discriminative OTUs identified in sPLS-DA as differentially enriched are marked with asterisks inside the graph.

The networks were dominated by the *Rumminococcaceae* and *Lachnospiraceae* families and were clustered by taxonomic families in the network graph. Airway obstruction phenotype groups showed a similar number of OTU nodes in the networks (λ index: 0.56 for the three groups) (Fig. 4A–C). However, the networks in FAO group were less complex (see Supplementary Fig. S6A) and interconnected as showed by a lower sparsity index (SI: 0.55) when compared with NAO (SI: 0.75) and RAO phenotypes (SI: 0.79); however, the strengths of edges were greater (see Supplementary Fig. S6B). Also, this network highlighted the enrichment of *Streptococcaceae:Streptococcus* (OTU546) and *Enterococcus:Escherichia-Shigella* (OTU92) and their difference in the connectivity compared to those networks derived from individuals in the NAO and RAO or NAO phenotypes (Fig. 4A–C and Supplementary Table S3).

### Several *Rumminococcaceae*, *Lachnospiraceae* and *Clostridiales* were enriched in patients with lower levels of IgE to Ascaris and HDM

**Figure 5:**
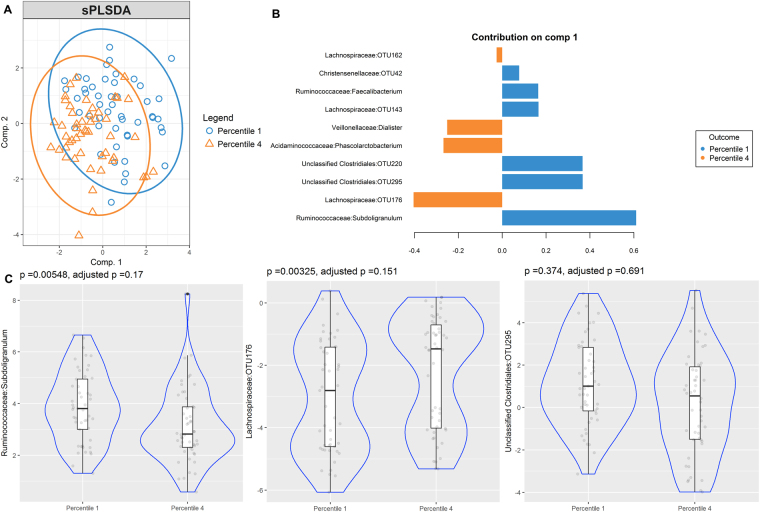
Partial least squares discriminant analysis of gut microbiome composition between low and high IgE responders to Ascaris. sPLS-DA plot based on the relative abundance of bacterial taxa of gut microbiota from low (blue circle, percentile 1) and high IgE responders (orange triangle, percentile 4) and their 95% confidence ellipses (**A**). Contribution plot indicating genera contributing to component 1 of the sPLS-DA plot that discriminate first and fourth sIgE percentiles (**B**). The abundance of the most consistent OTUs was compared using Metagenomeseq and presented on a violin plot, which includes the median, 95% CI, IQR, and density plot where the width of the blue lines indicate frequency (**C**).

**Figure 6:**
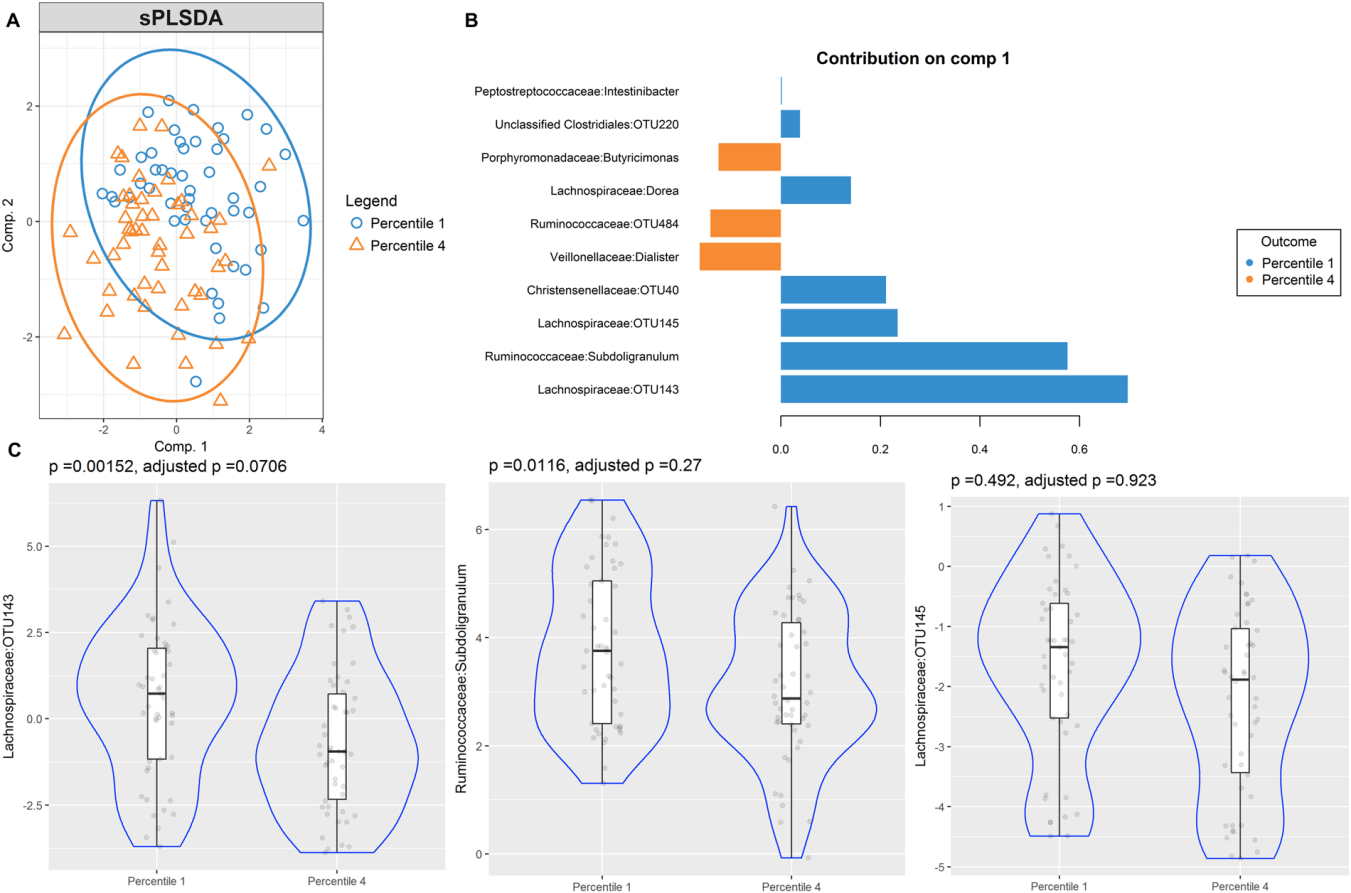
Partial least squares discriminant analysis of gut microbiome composition between low and high IgE responders to *B. tropicalis*. sPLS-DA plot based on the relative abundance of bacterial taxa of gut microbiota from low (blue circle, percentile 1) and high IgE responders (orange triangle, percentile 4) and their 95% confidence ellipses (**A**). Contribution plot indicating genera contributing to component 1 of the sPLS-DA plot that discriminate first and fourth sIgE percentiles (**B**). The abundance of the most consistent OTUs was compared using Metagenomeseq and presented on a violin plot, which includes the median, 95% CI, IQR, and density plot where the width of the blue lines indicate frequency (**C**).

**Figure 7:**
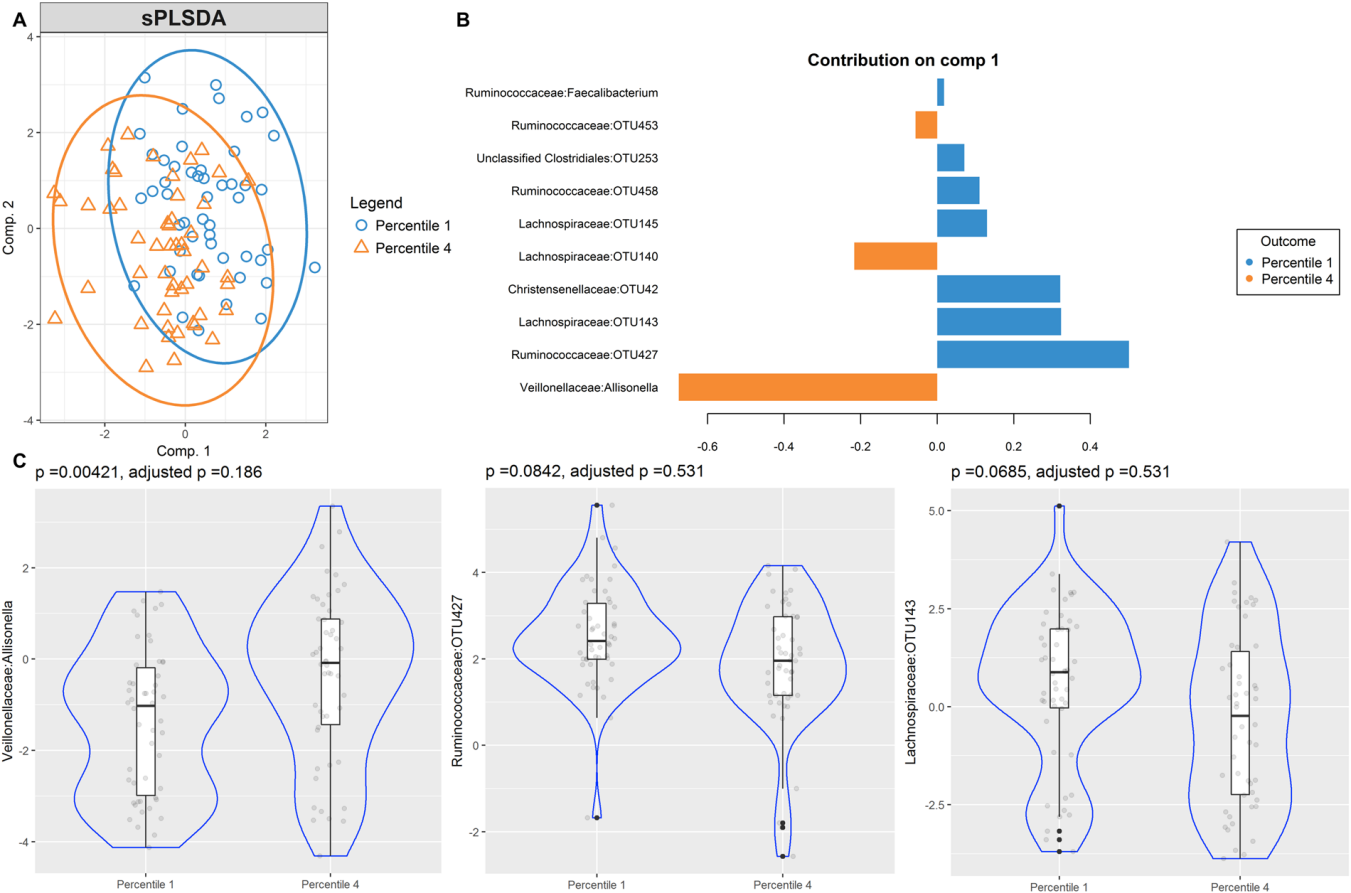
Partial least squares discriminant analysis of gut microbiome composition between low and high IgE responders to *D. pteronyssinus*. sPLS-DA plot based on the relative abundance of bacterial taxa of gut microbiota from low (blue circle, percentile 1) and high IgE responders (orange triangle, percentile 4) and their 95% confidence ellipses (**A**). Contribution plot indicating genera contributing to component 1 of the sPLS-DA plot that discriminate first and fourth sIgE percentiles (**B**). The abundance of the most consistent OTUs was compared using Metagenomeseq and presented on a violin plot, which includes the median, 95% CI, IQR, and density plot where the width of the blue lines indicate frequency (**C**).

Although cases of current Ascaris infection were scarce (n = 8), our analysis showed two groups according to infection status and various differentially enriched OTUs however it did not reach statistical significance after correction by multiple comparisons (see Supplementary Fig. S7). Gut microbiome of high and low specific IgE responders to Ascaris and HDM species, *B. tropicalis* and *D. pteronyssinus*, was also compared. The sPLS-DA bi-plots show discrimination of OTUs between low and high IgE responders to Ascaris (Fig. 5A), *B. tropicalis* (Fig. 6A) and *D. pteronyssinus* (Fig. 7A). Several OTUs supported those differences and were differentially enriched, contributing to the separation in the first component (Figs 5B, 6B and 7B). However, none of them kept significantly associated after adjustment by multiple comparisons (Figs 5C, 6C and 7C).

## Discussion

In this study, we compared gut microbiota composition among different airway obstruction phenotypes in asthmatic patients, representative of a socio-economically deprived population living in a tropical city. Although gut microbial richness and diversity was similar among phenotypes, there were differentially enriched microorganisms, being *Streptococcaceae:Streptococcus* and *Enterococcus:Escherichia-Shigella* more abundant in patients with FAO. In addition, a distinctive interconnected network enriched with those OTUs was characteristic.

A previous report by Hevia *et al*. comparing the gut microbiota composition between asthmatics and a healthy control group showed that patients with long term asthma harbour lower levels of *Bifidobacterium adolescentis* in the gut, but they did not found microbiota clustering according to asthmatic status, suggesting to some extent that globally gut microbiota in asthmatics is similar to healthy control subjects, except for the abundance of particular commensals^43^. Also, Hua *et al*. reported that the gut microbiota does not differ when compared between asthmatics and healthy controls according to α or β-diversity, but the identification of asthmatic category was not specific as included other lung disease^44^. The present study is the first evaluating associations between gut microbiota and the airway obstruction in a population of adult asthmatic individuals living in a tropical region. We sougth to explore differences in the gut microbiota composition explaining the airway obstruction phenotype in a population sharing the environmental exposure framework, looking for particular OTUs and their networks. In our study we found a consistent association between *Streptococcaceae:Streptococcus* and *Enterococcus:Escherichia-Shigella* with the FAO, and also *Veillonellaceae:Megasphaera* when comparing the FAO with RAO phenotype. Interestingly, a recent report by Arrieta *et al*. found an association between various *Streptococcus* and *Veillonella* species in the gut and the occurrence of wheeze in children from a low income tropical population at three months of age^22^ and at least for *Streptococcus spp*, associations for their increased abundance in the airway and wheeze^45,46^ or a severe asthma phenotype have been reported before^8^. Also, it has been shown that various species of *Streptococcus* and *Veillonella* populate the small intestine^47^ and appeared to block IL-12p70 production, while augmenting IL-8, IL-6, IL-10, and TNF-a responses by dendritic cells in *in-vitro* assays^48^, indeed these cytokines have been linked to airway obstruction and severe asthma phenotype^49–53^.

Although there is not a consensus definition for the FAO phenotype, it have been recognized as an asthma phenotype differentiated from COPD^54–58^ and explained by the progressive decline in FEV1 as a result of airway remodelling asscociated with allergic airway inflammation, allergen exposure and elevated IgE levels throughout life, in agreement with this is the fact that NAO patients were younger than the patients in the other phenotypes. As showed in other cohorts of patients^19,20^, in our study the FAO phenotype was not necessarily associated with more asthma symptoms, but a slightly increased prevalence of hospitalizations in the last year that deserves some mention. In contrast, in the RAO phenotype there was a stronger sIgE response to *D. pteronyssinus* and a tendency to more symptoms as described in other studies^17,59,60^, but a lower prevalence of hospitalizations, possibly those two different phenotypes of airway obstruction reflects two different clinical patterns of asthma. Smoking habit and exposure to cigarrete smoke can be aggravating factors, but they are not the only necessary condition for fixed airway obstruction outcome to occur in long lasting asthmatics^61^. As smoking habit and exposure to cigarrete smoke were uncommon and equally distributed across airway obstruction phenotypes in our population, it unlikely that those exposures explained the FAO phenotype. Instead, specific gut microbiota components appear as risks factors associated with the FAO phenotype, and this finding deserves more investigation employing more refined methodologies such as microbial whole genome sequencing to disentangle possible genetic pathways explaining the association.

In the other hand, NAO individuals showed an enrichment of various *Ruminococcaceae* and *Lachnospiraceae* when compared with the RAO or FAO phenotypes, but they were not significant after adjustment for multiple comparisons. Also, those belonging to the lowest sIgE responders to Ascaris and *B. tropicalis* showed enrichment of members from those two families plus various *Clostridiales* members. Various members of those families cooperatively metabolize the diet fiber and produce anti-inflammatory metabolites in the colon^62^ that decrease sIgE levels against *D. pteronyssinus* extract in a mice model of mite sensitization feeded with a high fiber diet^63^. On the other hand, gut microbiota signalling through TLR-5 and MyD88 dependent pathways regulates IgG and IgE levels against bacterial and non-bacterial antigens in mice^64–66^, also signalling through various pattern recognition receptor such as TLR-2 and TLR-4 has been associated with IgE mediated eccema^67^ and food allergy in humans^68^, suggesting that microbiota produced metabolites or specific structural components could stimulate innate immunity and influence systemic antibody levels. Although, after correction by multiple comparisons the abundance of commensal associated with protection was not significant, we speculate that protection against airway obstruction and sIgE levels could be accomplished by the combined presence of all those commensal as showed by sPLS-DA, but we can not assure causality or a specific mechanism.

As the gut comprises an extensive area populated with lymphoid organs and is highly vascularized and is proximal to the lung, the migration of dendritic, or another proinflammatory immune cells and cytokines induced by the microbiota to the lung is possible^69^, indeed previous findings in another inflammatory diseases such as arthritis and cerebrovascular diseases supports this possibility^70–72^. However, because of the cross sectional design of the study and the lack of an experimental animal model evaluating the effects of those commensals on allergic asthma, we cannot assure causality between the found taxa and airway obstruction phenotypes.

In our analysis we used sPLS-DA and network analysis assuming data to be compositional and sparse; in consequence CLR transformation was done, avoiding spurius association between some taxa and airway obstruction phenotypes. In undirected network construction, we used the SPIEC-EASI algorithm to robustly predict OTU-OTU interrelationships and construct a characteristic network for each phenotype. This algorithm begins with a neighborhood selection of commensals and selects those that better characterize the network (represented by the λ index) and calculate inverse covariance between them based on the concept of conditional independence to construct edge between nodes. The construction of edges is based on the abundance of commensals in the metadata for each patient belonging to a particular phenotype, remaining only with those conections that robustly reproduce in all patients after iteration and showing their number and weights represented by sticks connecting the nodes. Our results shows that OTUs clustered with another family related OTUs, as was described by Kurtz *et al*.^37^ for the analysis of fecal samples of healthy individuals belonging to the AGP. The fact that those OTUs discriminating between phenotypes of airway obstruction in SPLS-LDA (*Streptococcaceae:Streptococcus* and *Enterococcus:Escherichia-Shigella*) were also enriched in undirected network graphs supports our findings, as these two independent methods using compositinal data highlighted the importance of the same commensals and their networks.

Several limitations of this study should be mentioned. Although we only explored the association between gut microbiota composition and various clinical phenotypes of airway obstruction in asthma, the use of biological markers would have contributed to better characterize the severity of airway inflammation and its relationship with microbiome configuration. Although differences in age distribution among asthma phenotypes could introduce bias in the results, its relevance is low according to our sensitivity and age-OTU abundance association analyses. Also, dietary information and weight associated variables were not obtained. Corticosteroid use might also influence gut microbiome; however, in this study it was scarce and equally distributed between patients.

In summary, we found that *Streptococcaceae:Streptococcus* and *Enterococcus:Escherichia-Shigella* were consistently enriched in asthmatic individuals suffering a fixed airway obstruction in a socio-economically deprived population with poor controlled asthma sharing the environmental framework.

## Electronic supplementary material

Supplementary Dataset 1

Supplementary Dataset 2

Supplementary Dataset 3

Supplementary Figures
