# Supplementary material for: Gut microbiota components are associated with fixed airway obstruction in asthmatic patients living in the tropics
Source: Sci Rep. 2018 Jun 25;8:9582. doi: 10.1038/s41598-018-27964-3 (PMC6018556; doi:10.1038/s41598-018-27964-3)
Supplement: Supplementary file 4 — Supplementary Figures [file 41598_2018_27964_MOESM4_ESM.pdf]

SUPPLEMENTARY MATERIAL

Gut microbiota components are associated with fixed airway obstruction in asthmatic patients living in the tropics

Emiro Buendía, MD[1], Josefina Zakzuk, MD, PhD[1], Homero San Juan Vergara, MD, PhD[2], Eduardo Zurek, PhD[3], Nadim J. Ajami, PhD[4] & Luis Caraballo, MD, PhD[1]

[1]Institute for Immunological Research, University of Cartagena, Cartagena, Colombia. [2]Department of Medicine, Universidad del Norte, Barranquilla, Colombia. [3]Department of System Engineering, Universidad del Norte, Barranquilla, Colombia. [4]Alkek Center for Metagenomics and Microbiome Research, Baylor College, Houston, USA. *Correspondence to lcaraballog@unicartagena.edu.co

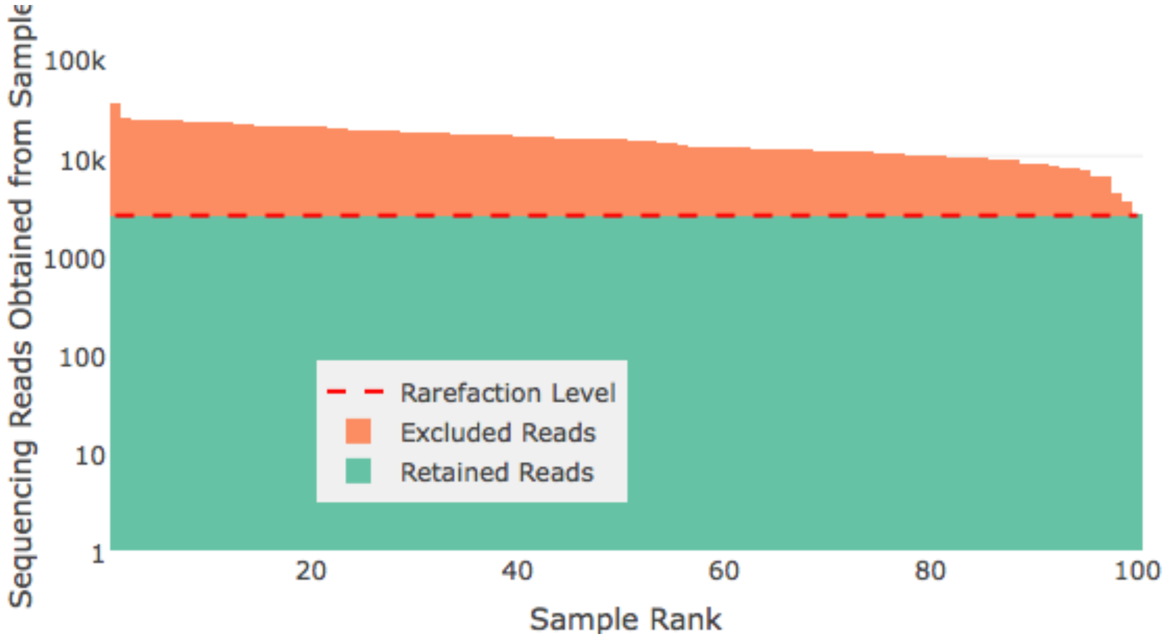

Supplementary Figure S1. Retained sequencing reads after rarification, setting a cutoff of 10.000 reads per sample.

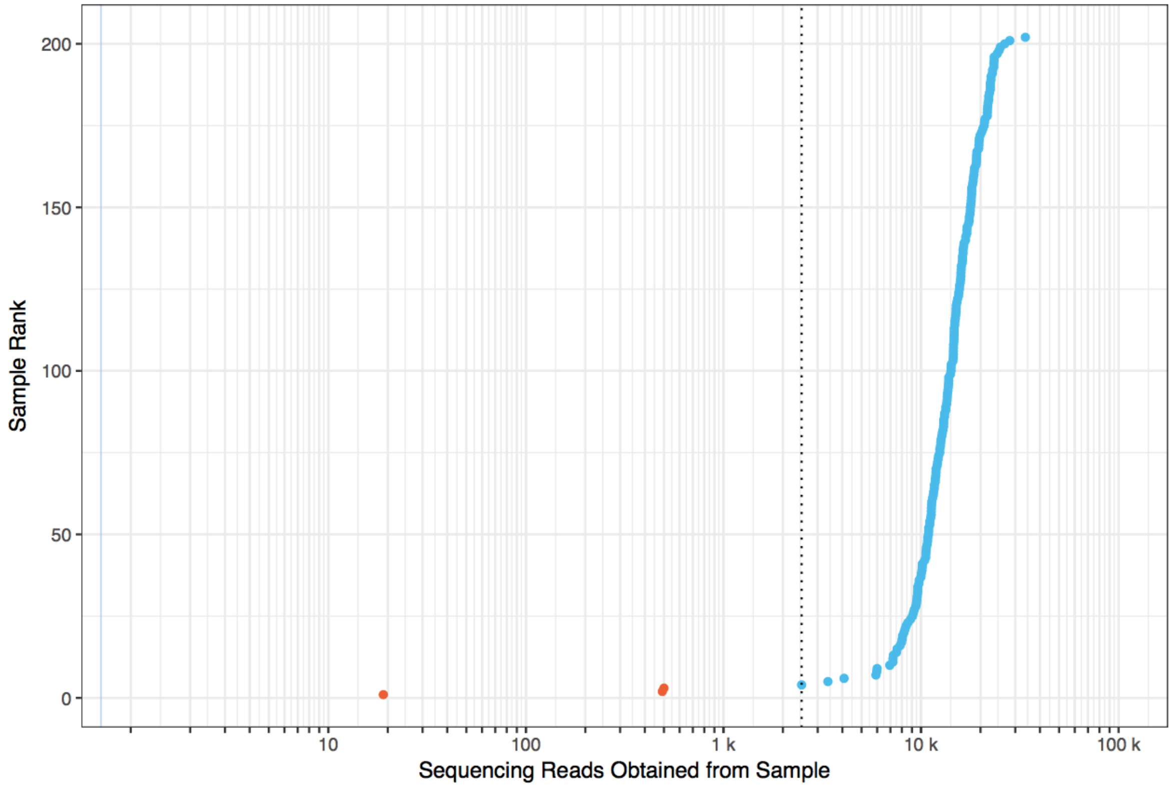

Supplementary Figure S2. Retained samples after after rarification, setting a cutoff of 10.000 reads per sample.

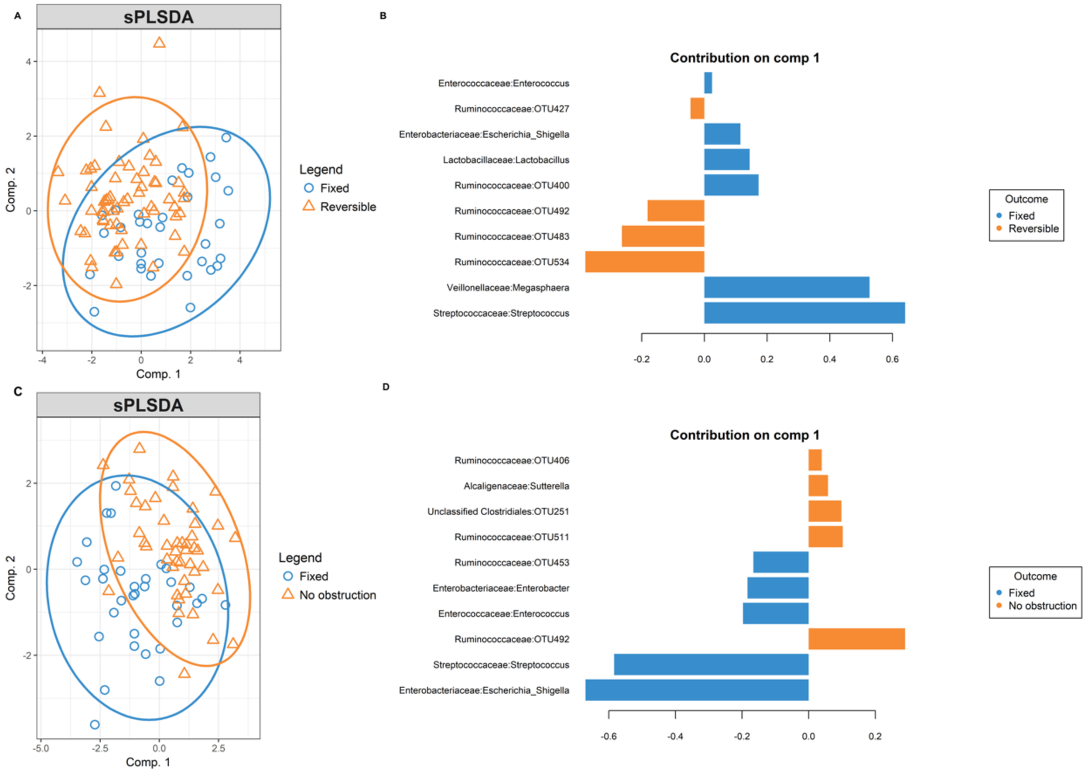

Supplementary Figure S3. Partial least squares discriminant analysis of gut microbiome composition between patients with fixed airway obstruction versus reversible or no obstruction excluding male patients. sPLS-DA plot based on the relative abundance of bacterial taxa of gut microbiota from patients with fixed airway obstruction (blue circle), reversible or without obstruction (orange triangle) and their 95% confidence ellipses (**A**). Contribution plot indicating genera contributing to component 1 of the sPLS-DA plot that discriminate these phenotypes (**B**).

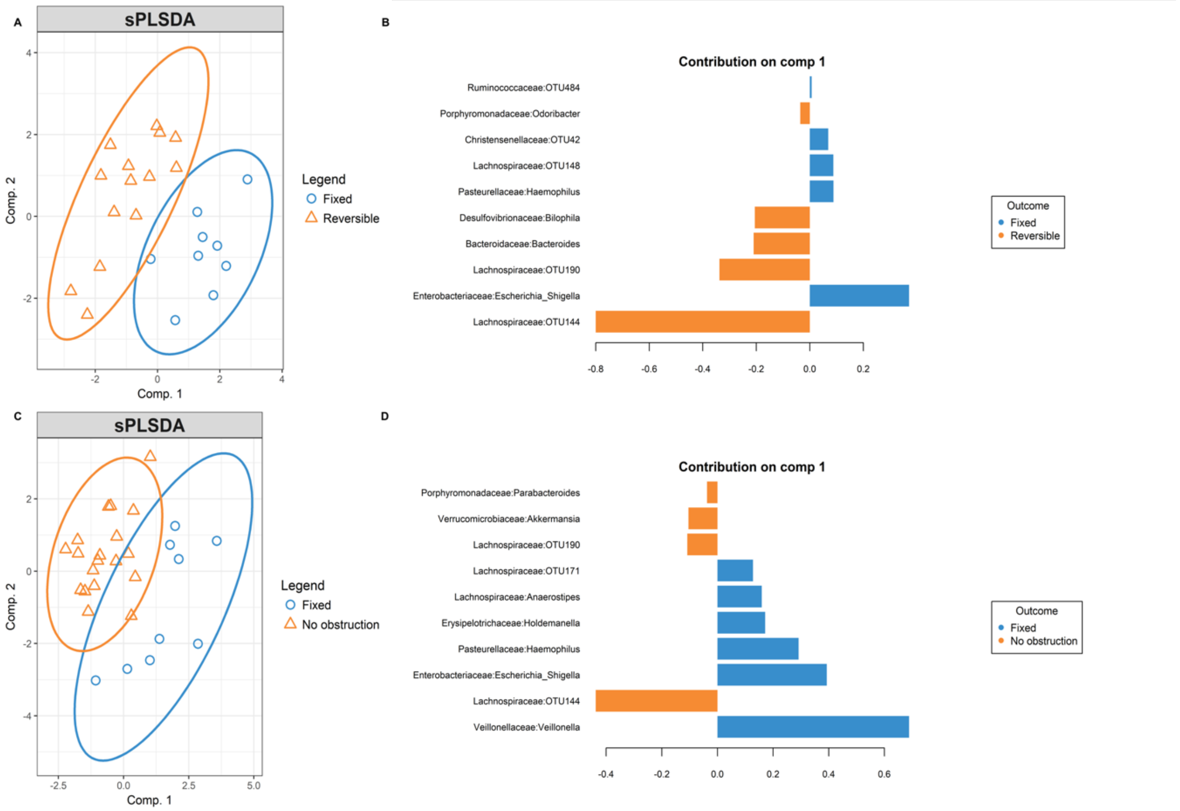

Supplementary Figure S4. Partial least squares discriminant analysis of gut microbiome composition between patients with fixed airway obstruction versus no obstruction excluding female patients. sPLS-DA plot based on the relative abundance of bacterial taxa of gut microbiota from patients with fixed airway obstruction (blue circle), reversible or without obstruction (orange triangle) and their 95% confidence ellipses (**A**). Contribution plot indicating genera contributing to component 1 of the sPLS-DA plot that discriminate these phenotypes (**B**).

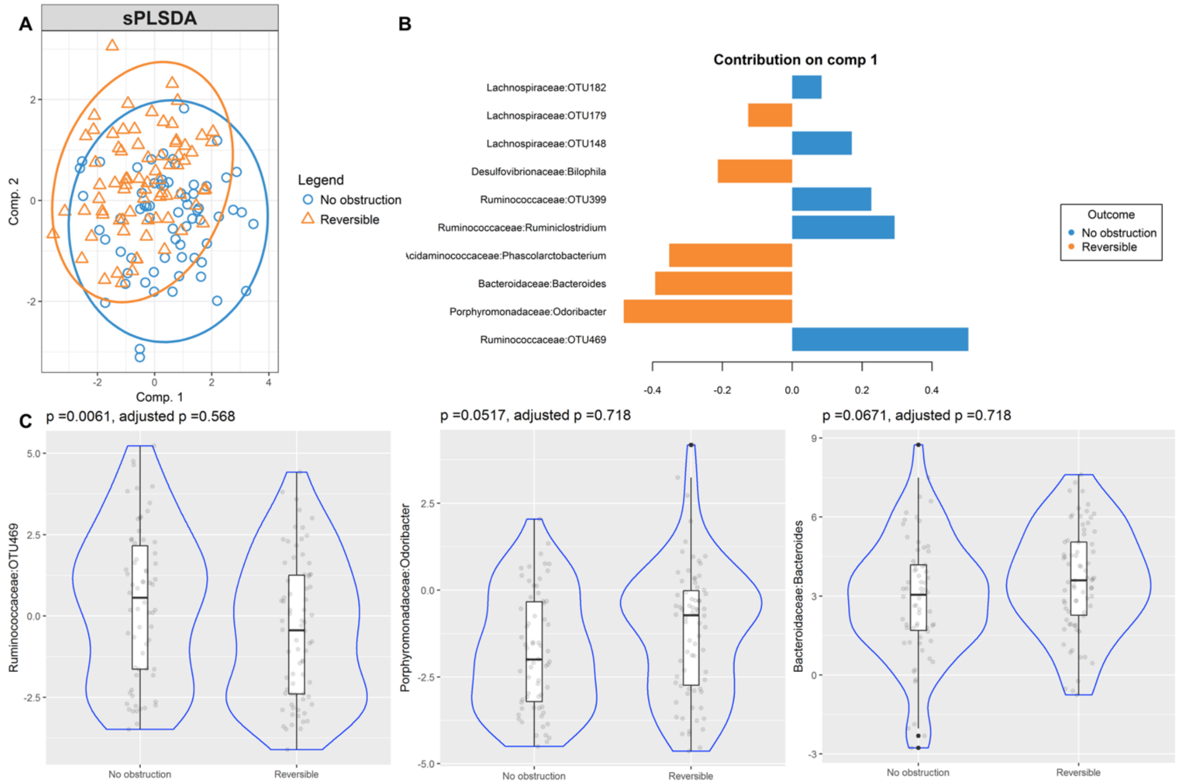

Supplementary Figure S5. Partial least squares discriminant analysis of gut microbiome composition between patients with no airway obstruction versus reversible obstruction. sPLS-DA plot based on the relative abundance of bacterial taxa of gut microbiota from patients with no airway obstruction (blue circle) or reversible airway obstruction (orange triangle) and their 95% confidence ellipses (**A**). Contribution plot indicating genera contributing to component 1 of the sPLS-DA plot that discriminate these phenotypes (**B**). The abundance of the most consistent OTUs was compared using Metagenomeseq and presented on a violin plot, which includes the median, 95% CI, IQR, and density plot where the width of the blue lines indicate frequency (**C**).

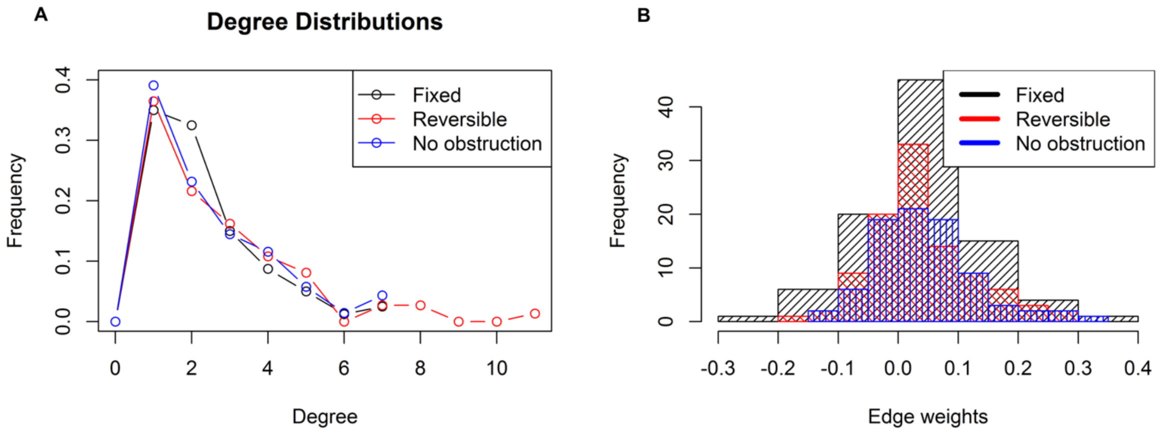

Supplementary Figure S6. Edge weights and degree distribution graphs. (**A**) Shows the distribution of connections per node. (**B**) Shows the frequency with which the weight of the arcs in each network occurs.

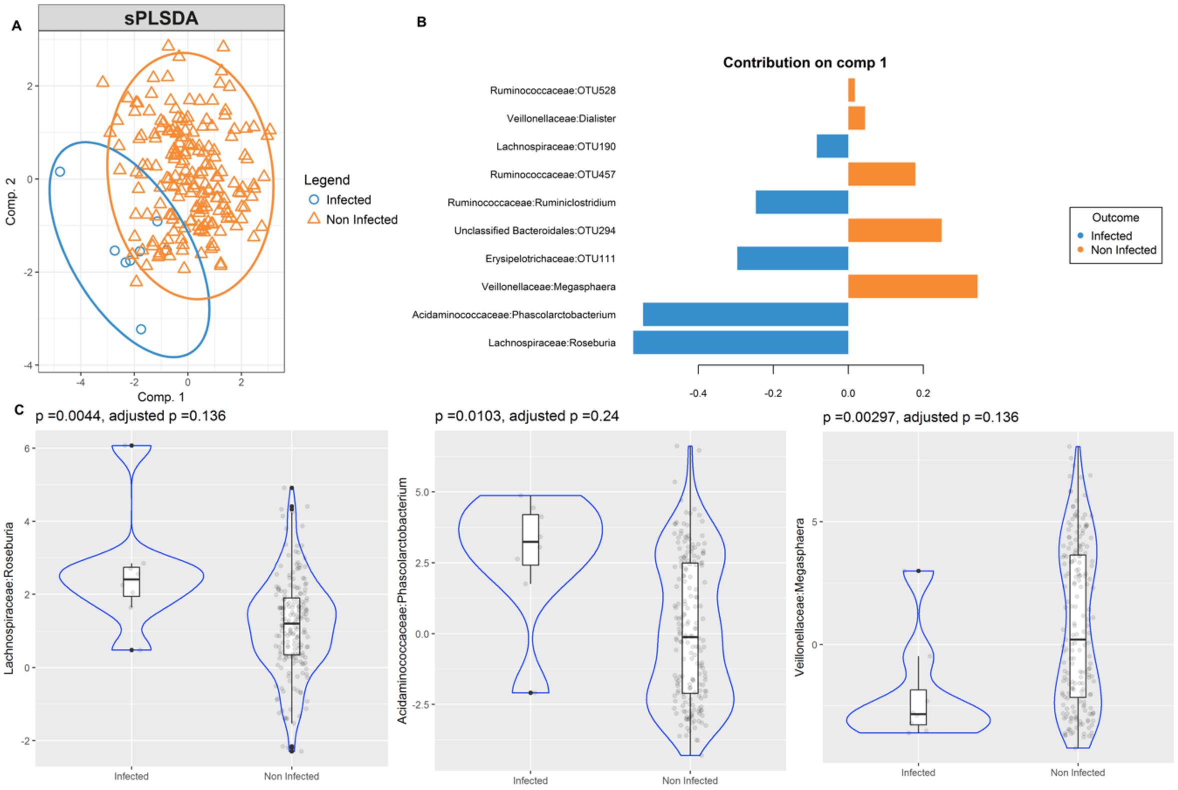

Supplementary Figure S7. Partial least squares discriminant analysis of gut microbiome composition in regard to Ascaris infection. sPLS-DA plot based on the relative abundance of bacterial taxa of gut microbiota from patients infected (blue circle) or not (orange triangle) and their 95% confidence ellipses (**A**). Contribution plot indicating genera contributing to component 1 of the sPLS-DA plot that discriminate first and fourth sIgE percentiles (**B**). The abundance of the most consistent OTUs was compared using Metagenomeseq and presented on a violin plot, which includes the median, 95% CI, IQR, and density plot where the width of the blue lines indicate frequency (**C**).
